# The Reversal of Drug-Resistance in Tumors Using a Drug-Carrying Nanoparticular System

**DOI:** 10.3390/ijms10093776

**Published:** 2009-08-31

**Authors:** Kyung Taek Oh, Hye Jung Baik, A Hyeong Lee, Young Taik Oh, Yu Seok Youn, Eun Seong Lee

**Affiliations:** 1 College of Pharmacy, Chung-Ang University, 221 Heukseok dong, Seoul 155-756, Korea; E-Mail:kyungoh@cau.ac.kr (K.T.O.); 2 Division of Biotechnology, The Catholic University of Korea, 43-1 Yeokgok 2-dong, Wonmi-gu, Bucheon-si, Gyeonggi-do 420-743, Korea; E-Mails:hinakira@naver.com (H.J.B.);mathcap3@naver.com (A.H.L.); 3 Department of Diagnostic Radiology, Yonsei University College of Medicine, Seodaemun-ku Shinchon-dong 134, Seoul, 120-752, Korea; E-Mail:oytaik@yumc.yonsei.ac.kr (Y.T.O.); 4 College of Pharmacy, Pusan National University, Jangjun-dong, Geumjeong-gu, Busan 609-735, Korea; E-Mail:ysyoun@pusan.ac.kr (Y.S.Y.)

**Keywords:** multi-drug resistance, nanoparticle, tumor treatment, drug delivery system

## Abstract

Medical applications of nanoparticular systems have attracted considerable attention because of their potential use in therapeutic targeting of disease tissues and their lower level of toxicity against healthy tissue, relative to traditional pharmaceutical drugs. The use of nanoparticular systems has been shown to overcome the limitations of most anticancer drugs in clinical applications. In particular, the improved performance of smarted nanoparticular system for solving the drug resistance problems that typically interrupt tumor treatment has provided a promising strategy for successful tumor chemotherapy. This review highlights recent studies that have examined the therapeutic effect of nanoparticular systems on drug-resistant tumors and presents insight on how they work.

## 1. Introduction

Cancer is a major worldwide public health problem. Currently surgery, radiotherapy, and chemotherapy are all used to treat different forms of cancer. They can each be used alone or together depending on a number of factors such as the type, location, and spread of the cancer [1,2]. In particular, chemotherapy still remains the primary modality for treating cancers. Controlling the treatment dose to balance effective anticancer activity and toxicity plays an important role in the success of chemotherapy [3,4]. However, one of the major problems with chemotherapy is damage to surrounding healthy organs and tissue because many anticancer drugs are designed simply to destroy cells. The threat of severe side effects caused by the random distribution of the drugs throughout the body has meant that maximum dosages must be restricted [5,6].

Another problem associated with the use of chemotherapy for cancer treatment is resistance against anticancer drugs [7–9]. Many types of wild cancers respond well to chemotherapy drugs in the beginning but show acquired resistance later [7–9]. The resistances of cancers that have been exposed to one cytotoxic agent develop cross-resistance to a whole range of drugs with different structures and cellular targets [7–9]. Once resistance appears, using higher drug doses to overcome resistance is ineffective because serious toxic effects appear and resistance is further stimulated [7–9].

It has been established that general nanoparticular systems can be used to decrease the non-specific toxicity of anticancer drugs by ‘hiding’ the drug in the core of the nanoparticular system and controlling drug uptake in normal tissues, which is similar to the ‘Trojan horse’ concept [11–13]. However, their therapeutic effects in regards to drug resistance were not significant.

A solution to overcome the problems of chemotherapy is the development of more advanced drug delivery systems for anticancer agents [10] that aim to improve the therapeutic efficacy for drug resistant tumors and patient compliance, and reduce toxic/side effects. In this review, several examples of advanced nanoparticular systems that have been developed to overcome drug resistance in tumors, which is a major hurdle for successful chemotherapy, are described.

## 2. Drug Resistance in Tumors

### 2.1. Multidrug Resistance (MDR) in Cells

**Figure 1.**
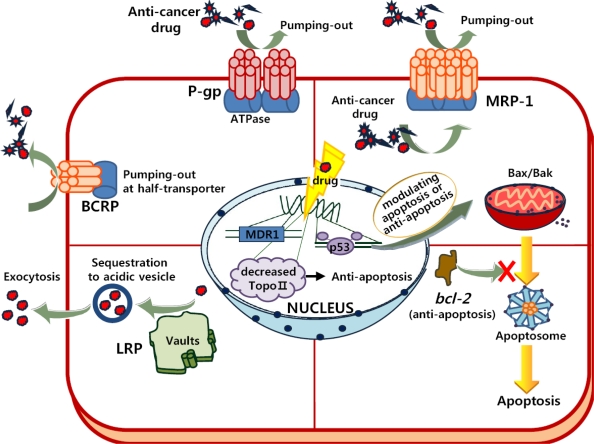
Drug-resistance mechanisms (such as P-gp, MRP, BCRP, LRP, p53, *bcl*-2, and TopoII) in tumor cells. See text for details.

Drug resistance in tumors can occur due to both impaired drug delivery to the cancer tissue and the defense mechanisms of the cancer cell itself [14]. In general, broad-based drug resistance, either intrinsic or acquired, exists in tumors and is believed to be caused multifactorially (Figure 1). This has significantly hindered clinical efforts to formulate effective chemotherapy strategies against several blood cancers, as well as solid cancers associated with breast, ovarian and lower gastrointestinal tract cancers [15–18]. Until recently, various tissue culture studies have consistently shown that MDR in most cultured cancer cells involves ATP-binding cassette (ABC) transporters in the human such as P-glycoprotein (P-gp, ABCB1) [19–21], multidrug resistance protein (MRP, MRP-1/ABCC1) [22–24], breast cancer resistant protein (BCRP, ABCG2) [25], lung resistant protein (LRP) [26–28], *bcl*-2 [29], p53 [30,31], Topoisomerase II (TopoII) [32,33], etc.

P-gp, which is encoded by the MDR1 gene, is an ABC transporter normally involved in the excretion of toxins from cells using energy from ATP hydrolysis [19–21]. Generally, P-gp is expressed in normal tissues (such as immune-response cells, epithelial cells of colon, kidney, adrenal, pancreas, and liver) as part of the defense mechanism of the body [19]. When chemotherapy agents cause DNA damage in tumor cells, P-gp is over-expressed due to the activation of MDR1/ABCB1 (one of two isoforms of P-gp). Cells that over-express P-gp in the cellular membrane have been reported to display a strong drug resistance against a whole range of lipophilic drugs [20–21]. MRP-1 or BCRP is another member of the ABC transporter [22–25]. They are located on the cellular membrane or cytoplasmic vesicles and appear to transport lipophilic or anionic drugs outside of cells [22–24]. Although the hydrophobic vacuum cleaner model was proposed to evacuate drugs due to the interaction of P-gp with drugs partitioned into bilayer [24], the mechanism behind this function has not yet been well established. LRP is localized in cytoplasmatic vaults for sequestration of anticancer drugs into acidic vesicle from the cytoplasm [26–28]. Most of the vaults are located in the cytoplasm, but a portion of the vaults is present in the nuclear membrane or nuclear pore complex [26]. Due to the probability of LRP localization in these vaults, LRP has the ability to transport substrates from the nucleus to the cytoplasm. Thus, the sequestered drugs could not promote DNA damage [27,28]. Furthermore, LRP can remove anticancer drugs from cells via exocytosis of acidic vesicles containing the trapped anticancer drugs [26,27]. In addition, LRP and P-gp might share a similar regulatory mechanism mediated by p53 [28].

Apoptosis, or programmed cell death (PCD), is a cellular self destruction mechanism involved in a variety of biological events, such as developmental sculpturing, tissue homeostasis, and the removal of unwanted cells [29–31]. Many anticancer drugs potentially induce PCD [1–5]. The balance of factors promoting and protecting PCD (*e.g.*, p53, *bcl*-2, Bax/Bak) is a decisive component regulating the intrinsic chemosensitivity of a cell [29–31]. Resistance to PCD induced by chemotherapeutic drugs is assumed to involve mainly the over-expression of *bcl*-2 family members and the loss of wild-type p53 [30]. When *bcl*-2 (anti-apoptosis factor) is over-expressed, the formation of apoptosome (consisting of Apaf-1, cytochrome c, and caspase) in drug-treated tumor cells may be blocked by the following cellular responses: *i*) inhibiting the release of cytochrome c from the mitochondria and preventing binding of cytochrome c and Apaf-1, *ii*) directly combining with Apaf-1, *iii*) directly binding several caspases and preventing caspase activation [29]. In addition, the DNA-binding transcription factor p53 medicates a block of cell cycle entry into S phase in order to repair DNA damages and to activate a PCD pathway for serious DNA damages. Thus, the loss of wild-type p53 leads to both genomic instability and resistance to PCD by activating Bax/Bak (pro-apoptotic factors) from the mitochondria, which prevents the cell death process from occurring [30,31].

Topoisomerases (Topo) are essential nuclear enzymes that can change the topological state of the DNA by breaking and rejoining the DNA phosphodiester backbone. Many anticancer agents have targeted Topo II [32]. When DNA is damaged by anticancer drugs, Topo II also breaks DNA double helices and promotes the formation of a cleavable complex that ultimately leads to cell death [32]. However, a reduction in the level of Topo II in MDR tumors due to the aberrant transcript by mutations, and posttranslational modification, cells leads to a decrease in the formation of the cleavable-complex, thereby preventing apoptosis of tumor cells [32,33].

### 2.2. Drug-Resistance in Microenvironment of Tumors

**Figure 2.**
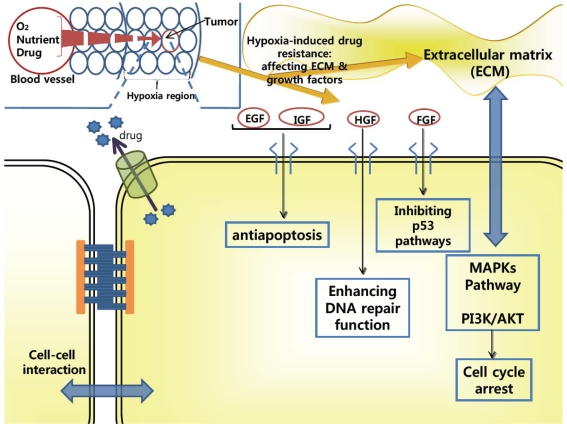
Drug-resistance mechanisms (such as soluble growth factors, ECM-based drug resistance, cell-cell interaction, and hypoxia-induced drug resistance) in the tumor microenvironment. See text for details.

Drug resistance due to the microenvironment of tumors is one of the most important obstacles to tumor treatment (Figure 2). In clinical tumors, it is difficult to deliver oxygen and other nutrients to tumor cells because the tumor cells are located in a poorly organized vasculature and are far from blood vessels [34]. This hypoxic region is resistant to chemotherapy because drug penetration into this region is very limited [34]. Since tumor cells in this hypoxic region are non-proliferating or slowly proliferating with increasing distance from tumor blood vessels, most anticancer drugs are less active [34]. It has been reported that this region modulates the elevation of anti-apoptosis proteins to prevent cell death and the up-regulation of growth factors for cell growth [35]. In particular, several growth factors such as epidermal growth factor (EGF), fibroblast growth factor (FGF), insulin-like growth factor (IGF), and hepatocyte factor (HGF), have important functions in regards to MDR, cell proliferation, metastasis, and angiogeneis [35,36]. These growth factors are closely related to chemoresistance, although there are several reports that these growth factors conversely enhance the chemosensitivity [37–39]. As shown in Figure 2, binding of EGF, FGF, and IGF to their receptors leads to up-regulation of anti-apoptotic proteins (*bcl*-2 family members such as *bcl*-2, *bcl*-XL, and LAPs), resulting in inhibiting apoptosis. In addition, binding of FGF to its receptor mediates obstructing p53 pathways. HGF binding to its receptor enhances the DNA repair function, which is related to anti-apoptotic function [35–39].

On the other hand, altered expression of extracellular matrix (ECM) components (such as fibronectin, collagen, tenacin, laminin, and hyalurona) contributed to protecting tumor cells from anticancer drugs, by activation of MAPKs and PI_3_K/AKT survival signaling, decreasing TopoII level, and arresting cell proliferation due to the increased cyclin-dependent kinase (CDK) inhibitor p27/Kip1 protein [39–41]. In addition, cadherins, selectins, and cell adhesion molecules (CAMs) can make cell-cell contact and exhibit further drug-resistance [41,42].

Most importantly, the role of the microenvironment in the drug-resistance of tumors is multi-factorial [34–42]. Growth factors modulate the drug-resistance of cancer cells and usually activate changes in adhesion molecules [35–38]. The adhesion molecules such as Cadherin promote the signaling of soluble factors, which induce the anti-apoptotic factors [39–42]. Tumor cells adherent to ECM components attenuate growth factor-mediated cancer cell protection [39–42]. Hypoxia increases anti-apoptotic proteins (*e.g.*, *bcl*-2, *bcl*-XL, IAP family members), arrests cell cycle (due to increased CDK inhibitors p27/Kip1 and p21/Clip1), and elevates glutathione S-transferase-π level (associated with tumor progression and invasion). In addition, hypoxia through hypoxia-induced factor-1 (HIF-1) exhibits the expression of ATP-binding cassette drug effluxes (P-gp and BCRP). On the other hand, hypoxia modulates the tumor microenvironment by up-regulating vascular endothelial growth factor and its receptors, and facilitates aggressive proliferation of tumor cells with genetic instability [34,35].

## 3. Overcoming Drug-Resistance

### 3.1. Using P-gp Modulators

The identification of verapamil as a P-gp blocking agent inspired numerous investigations into discovering MDR inhibitors [43]. Various chemo-agents such as cyclosporine A, glibenclamide, PSC833, GF120918, XR9576, LY335979, etc have been developed to overcome MDR phenotypes in tumors [43–48]. Studies on minimizing pharmacokinetic interactions with anticancer drugs have also been conducted [49]. However, these chemo-agents have not yet been effective in Phase III trials [43–49]. In most cases, the ability of P-gp blocking chemo-agents to prevent the action has been examined in *in vitro* tumor cells, even though quite a few clinical trials involving P-gp reversal agents [43]. However, MDR in *in vivo* cancer cells results from multiple drug-resistant mechanisms and it is not caused by just P-gp [43–49]. P-gp blocking chemo-agents can also interact with the P-gp of healthy organs such as placenta, kidney, liver and kidney, resulting in more toxic effects of a given anticancer drug [43–50]. PSC833 (P-gp blocking chemo-agent) failed Phase III trials [50] because of these causes. Kabanov’s group has used Pluronic^®^ block copolymers, instead of chemo-agents, to interrupt the P-gp mediated drug efflux pump [51,52]. Pluronic formulations with anticancer drugs, below their critical micelle concentration (CMC), have been claimed to be effective in treating MDR tumors [51,52]. These results have been linked to the ability of Pluronic^®^ (PEO-PPO-PEO) block copolymers to cross the plasma membrane and suppress ATP production, although the mechanism of this function is still unknown. This effect has also been linked with gene modulation by Pluronic^®^ block copolymers [51,52]. This formulation seems to be effective with MDR tumors but interestingly it is less effective with wild tumors [51,52]. In addition, Pluronic formulations lack tumor specificity and not much is known about its influence on normal cells expressing P-gp.

### 3.2. Using Nanoparticular Systems

Many groups have studied the ability of a variety of nanoparticular systems to overcome MDR for tumor treatment (Table 1). Compared to conventional chemo-therapy, nanotherapeutic systems have several potential advantages for cancer treatment, including easy modification of particle surface for targeting systems, increased stability in blood, dual delivery such as drug, gene, and/or imaging agents, drug delivery system responding to environmental stimuli such as temperature, pH, salt, and ultrasound, etc. These systems include liposomes, polyalkylcyanoacrylate nanoparticles, polymeric micelles system, etc [53–76].

First, one strategy using nanoparticular systems to overcome the MDR cancers is to formulate both anticancer agents and biological modification agents (such as P-gp inhibitors, ATP depletion molecules, and cell membrane modifiers) into nano-systems. These systems primarily accumulate passively in solid tumors by a process termed ‘enhanced permeability and retention’ (EPR) effects [54]. Lu *et al*. have formulated topotecan (anticancer drugs) and amlodipine (P-gp blocking chemo-agent) into stealth liposomes [53]. It is known that amlodipine blocks Ca^2+^channel through activation of caspase 8, caspase 3, and caspase 7, decreases intracellular Ca^2+^ level, and acts as a substrate of P-gp [53]. First, this system has been shown to result in an accumulation in solid tumors by EPR effect. This system may then provide increased anticancer activity due to the P-gp inhibition of amlodipine [53]. Similarly, other groups have designed liposome/Pluronic^®^ F68 or liposome/verapamil systems, involving encapsulated doxorubicin (anticancer drug) [55,56]. These trials presented enhanced cellular uptake of nanoparticular systems due to pinocytosis and retention of anticancer drugs in *in vitro* tumor cells, followed by ATP depletion by Pluronic^®^ block copolymer or direct binding of verapamil to P-gp on specific sites [54,56]. However, it was also noted that blocking MDR using these systems actually required a high dose, which can increase toxicity and affect the pharmacokinetics of the anticancer drugs [56,57].

Minko’s group has developed PEGylated liposomes with doxorubicin and antisense oligonucleotides to target *bcl*-2 and P-gp [58]. This antisense oligonucleotides delivery system was shown to simultaneously inhibit the pumping mechanism in MDR cells and substantially enhance tumor apoptosis in mice bearing a xenograft of human MDR ovarian carcinoma [58], although the tumor targeting ability was not optimal and the practical development of a cytosolic antisense oligonucleotide delivery system was not satisfied.

Couvreur’s group reported that polyalkylcyanoacrylate (PACA) nanoparticles with doxorubicin could reverse P-gp by modulating interactions between PACA nanoparticles and the cell surface [59,60]. However, the interaction mechanism of PACA nanoparticles with tumor cells remains unclear. Overall, it is questionable whether these investigations, which target a particular MDR type (such as P-gp), will be effective against *in vivo* MDR cancers that involve various MDR phenotypes.

Recently, Au’s group introduced another strategy such as a tumor priming technique to enhance the anticancer delivery and efficacy of chemotherapy [61]. As mentioned above, the anticancer drugs are not effectively delivered into the hypoxia region due to the high density of solid tumors in clinic situation. They pretreated tumors with anticancer drugs, leading to the reduced cell density. Subsequent drug treatment allowed for drug penetration into the inner layers of a solid tumor. This tumor pretreatment (tumor priming) with paclitaxel (PAC) expanded the interstitial space and vessel diameter around tumors, increasing the doxorubicin-loaded liposomes (Doxil^®^)’ anticancer activity and long-term survival rate [61]. This test suggests a potentially useful means to enhance the degree of tumor penetration by the nanoparticular system, even if the tumors exist in a hypoxic condition. However, the systems are in doubt to overcome MDR phenotypes related to molecular mechanisms such as ABC transporters.

Rapoport *et al*. have observed that a drug-loaded polymeric micelle system could efficiently delivery anticancer drugs to wild and MDR tumors using an ultrasound technique [63,64]. The ultrasound leads to the internalization of micelles rather than inducing mechanical damage to the cellular membrane [63,64]. Immediate cell killing by the ultrasonic impact was not observed in their experiments. Tumor cells were readily killed by the cytotoxic activity of the drug released from the micelles after they were internalized [63,64].

On the other hand, several groups have tried to overcome the MDR by employing a drug delivery system modified with active targeting moiety. The systems could be expected to increase the intracellular drug concentration by avoiding the P-gp pathway through active internalization (*e.g.,* receptor-mediator endocytosis) of the drug-loaded carriers. Fang *et al*. have studied taxol-loaded mixed micelle systems with Pluronic^®^ block copolymer P105 or L101 [65]. This system was modified with folic acid to target a variety of tumor cells over-expressing the folic acid receptor (FR). It is beneficial to concentrate drug-loaded micelles into solid tumors with FR [65,66]. However, the drug release from micelle into cytosol or nucleus was in doubt and there is a possibility of exocytosis, another MDR mechanism. Similarly, Kiwada *et al*. have reported transferrin receptor-targeting liposomal doxorubicin [67]. This formulation was anticipated to bypass P-gp over-expressed on cellular membranes because the internalization pathway, receptor-mediated endocytosis, is independent of the P-gp pathway. Alberto Gabizon *et al*. have also used the liposomal system with folic acid [68]. However, no significant tumor-growth obstruction was observed in these naoparticular systems, which may have been due to inefficient cytosolic anticancer drug release and lower drug concentration in the target sites such as the nucleus and mitochondria.

Overall, these investigations have showed limited success in the states of *in vitro* or preclinic study. They have usually targeted a single MDR mechanism in tumors associated with various MDR mechanisms. As a result, no approach has been proven to be effective in clinical MDR tumor treatments so far.

### 3.3. Smart Nanoparticular Systems

For the reversal of complicated mechanism in MDR, smart nanoparticular system has been developed with the concept of ‘bunker buster’ using endosomal pH (<pH 7.0) targeting systems [69–76]. The smart nanoparticular system composed of targeting moiety and pH-sensitive blocks, effectively transport anticancer drugs into cytosol without detection of ABC transporters (due to receptor-mediated entocytosis of system) and with the breaking of endosome. The breaking of endosome is responsible for ‘proton sponge effect,’ which arises from a large number of weak conjugate bases (with buffering capabilities at pH 5–6), leading to proton absorption in acid organelles and an osmotic pressure buildup across the organelle membrane [77].

Lee *et al*. have designed poly(l-histidine) (polyHis, Mw 5KDa)-*b*-poly(ethylene glycol) (PEG, Mw 2KDa) based nanoparticular micelle system with pH-responsive properties [69–72]. The polyHis is lipophilic and becomes deprotonated when the pH is above the pK_b_, while PEG is soluble in water at all pHs [69]. This amphiphilicity was responsible for the formation of self-assembly polymeric micelles [69–72]. Lowering pH of the solution below the pK_b_ destabilized the micellar core structure due to that protonation of the polyHis [70]. Consequently, this micelle was disintegrated below pH 7.2 due to the protonation of the polyHis block forming the micellar core [69]. The mixed micelle system, which was composed of polyHis-*b*-PEG (75 wt %) and poly(l-lactic acid) (PLLA, Mw 3KDa)-*b*-PEG (Mw 2KDa) (25 wt%) block copolymers decorated with folic acid (for FR-mediated tumor targeting), presented excellent colloidal stability at pH 7.0–7.4, but destabilized below pH 7.0, resulting from the incorporation of a non-ionizable block copolymer (PLLA-*b*-PEG) [70]. Interestingly, the mixed micelles were able to active intracellular translocation of the drug-carriers via specific interactions such as FR-mediated endocytosis by escaping pumping out the administrated drugs by P-gp. The systems translocated as a formation of endosome could trigger drug release at endosomal pHs (pH < 7.0) [70,71]. The proton-sponge effect of polyHis also modulated endosomal disruption for cytoplasmic drug release [70,71]. These properties of the polyHis-based micelle system resulted in an enhanced drug concentration in cytoplasm or nucleus, which were successful in killing *in vitro* and *in vivo* tumor cells over-expressing P-gp [70,71]. The biodistribution of this system showed the more accumulation of drug in the MDR tumor compared to that of free drug [71].

**Figure 3.**
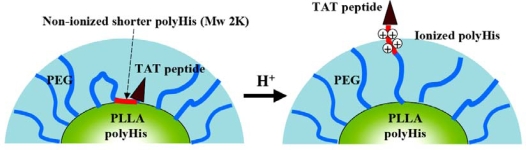
Schematic representation of the acid-induced pop-up targeting mechanism (PHSM^pop-upTAT^) of the peptide-conjugated micelle corona. See text for details. Reproduced with permission from reference [73].

On the other hand, Lee *et al*. have developed multifunctional pH-responsive polymeric micelle system (denoted as PHSM^pop-upTAT^) for treating various MDR phenotypes [72,73]. Figure 3 describes the central concept of this polymeric micelle system with pH signals. The PHSM^pop-upTAT^ consisted of polyHis (Mw 5Kda)-*b*-PEG (Mw 3.4Kda) and PLLA (Mw 3KDa)-*b*-PEG (Mw 2KDa)-*b*-polyHis (Mw 2KDa)-TAT peptide. The shorter polyHis block (Mw 2KDa) was located at the interface of the micellar core [consisting of longer polyHis (Mw 5KDa) and PLLA] and TAT peptide block conjugated with shorter polyHis block was simultaneously buried in the PEG forest (hydrophilic shell). However, as the pH was lowered below pH 7.0, the degree of ionization of the shorter polyHis block increased and the TAT peptide block was gradually exposed to the outside of the hydrophilic shell [73]. It is known that the TAT peptide (non-specific cell penetrating peptide derived from human immunodeficiency virus type 1 and 2) serves to translocate nanoparticles into cells due to the energy-dependent endocytosis (or macropinocytosis) after electrostatic interaction [74]. The TAT peptide exposed on the surface of the micelle can provide active internalization of mixed micelles into cells, regardless of the broad heterogeneity of tumor cells. As a result, this system showed high accumulation of anticancer drugs in tumor cells and exhibited cytosplasmic drug release due to the proton sponge effect of longer polyHis block dissociated from PHSM^pop-upTAT^ at an endosomal pH [74]. These process sharply elevated the drug concentration to levels much higher than the cytotoxic threshold dose in tumor cells and helped to eliminate various MDR phenotypes (such as MRP, LRP, *bcl*-2 and TopoII) (Table 2) [73]. The IC50 of PHSM^pop-upTAT^ and the free drug is shown in Table 2. Furthermore, a novel virus-like nanogel (virogel) system that has infectious properties (just like virus) for wild and MDR tumor cells was developed based on the findings of this system [75].

**Figure 4.**
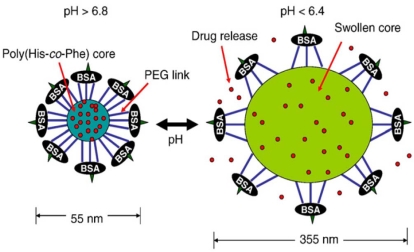
Schematic presentation of the virogels. See text for details. Reproduced with permission from reference [76].

The virogel system is now one of the most promising nanoparticular delivery systems because this system has cell specific infection, injects a toxin, destroys MDR tumor cells, and migrates to neighboring MDR tumor cells with repeated cycles [75]. This system consists of a lipophilic core [poly(l-histidine-*co*-phenylalanine): poly(His-*co*-Phe)] and two hydrophilic shells [PEG and bovine serum albumin (BSA)]. One end of the PEG is linked to the core-forming block and the other is randomly linked to BSA, which forms a capsid-like shell (Figure 4) [76]; this structure is formed by an Oil-in-water emulsion method. It is worth noting that this system has a reversible swelling/deswelling property that is dependent on pH. The anticancer drug (doxorubicin) is released when the virogel is swelled at pH 6.4 (endosomal pH), but the rate of drug release is reduced when the virogel is in the deswelled state at pH 7.4–6.8 (cytoplasmic pH). Moreover, this system can physically disrupt the endosomal membrane due to the volumetric expansion (average particle size ~355 nm at pH 6.4) of the virogel at endosomal pH. Thus, upon endosomal uptake and acidification the virogels release the drug and disrupt the endosomal membrane. The virogels then shrink in the cytoplasm in response to the cytoplasmic pH, and wait lysis of the tumor cell due to the release of the anticancer drugs in the endosome. The virogels then are released from the lysed cell and can subsequently infect neighboring tumor cells. Although further *in vivo* investigations are required, this nanoparticular system is believed to be a highly promising candidate for treating wild and MDR tumors.

## 4. Conclusions

Researchers have revolutionized the nanoparticular system for tumor treatment, especially in regards to overcoming MDR phenotypes in tumors. Nanosystems were tailor-made corresponding to the pharmaceutical demands, improved therapeutic effectiveness due to the combination therapy with multiple drugs. Some nanoparticular systems decorated with endogenous ligands, were internalized into tumor cells due to receptor-mediated endocytosis; thus, these systems were advantageous in that they bypassed P-gp over-expressed on the cellular membrane. Furthermore, the pH-responsive micelle systems were capable of achieving controlled drug release and endosomal escape, which were both vital in overcoming various MDR factors.

Although several attempts at overcoming MDR have been studied in the various areas of cancer research, the results are not yet satisfied in clinical situation. Of course, it is not an easy task to treat MDR tumors associated with various MDR phenotypes. Nevertheless, these nanoparticular systems are expected to continually promote the creation of novel strategies for treating MDR tumor cells and will be instrumental in the development of novel chemo-agent.

## Figures and Tables

**Table 1.** Reversal of drug-resistance by nanoparticular systems.

**Nanoparticular formulation**	**Drug-resistance target**	**Feature**	**Ref.**
Pluronic^®^ micelle with ultrasound treatment	Enhancing drug uptake by ultrasound treatment	Possible to treat wild and drug-resistant tumors	[63,64]
Paclitaxel loaded mixed micelle system of Pluronic^®^ P105 and L101	Inhibition of P-gp by Pluronic^®^	Combined mechanisms of FR-mediated endocytosis for tumor targeting	[65]
Liposomal formulation with doxorubicin/paclitaxel/valspodar	Inhibition of P-gp by valspodar	-	[44]
Liposomal topotecan with amlodipine	Inhibition of P-gp by amlodipine	-	[52]
Liposomal doxorubicin/verapamil	Inhibition of P-gp by verapamil	Verapamil affected pharmacokinetics of doxorubicin *in vivo*	[56]
Liposomal doxorubicin/Pluronic^®^ F68	Inhibition of P-gp by Pluronic^®^	-	[54]
Liposomal doxorubicin/antisense oligonucleotides	Targeted to *bcl*-2 mRNA and MDR1 mRNA	Overcoming *bcl*-2 and P-gp	[58]
Polyalkylcyanoacrylate nanoparticles with doxorubicin and cyclosporin A	Enhancing drug uptake by unknown mechanisms of polyalkylcyanoacrylate nanoparticles	Cyclosporin A can affect pharmacokinetics of doxorubicin	[59,60]
Daunorubicin loaded Fe_3_O_4_ nanoparticles	Enhancing drug uptake by Fe_3_O_4_ nanoparticles	Interaction between Fe_3_O_4_ and unknown biological active molecules on the membrane of leukemia cells, increased drug uptake	[74]
Poly(ethylene oxide)-modified poly(epsilon-caprolactone) (PEO-PCL) nanoparticle with ceramide and paclitaxel	Targeting to P-gp	Co-therapy (ceramide and paclitaxel) enhanced cytotoxicity for drug-resistant tumors	[62]
Transferrin receptor-targeting liposomal doxorubicin	Evading P-gp function by transferring receptor-mediated internalization pathway	-	[67]
Folate-conjugated liposomal doxorubicin	Evading P-gp function by FR-mediated internalization pathway	No significant tumor-growth inhibition effect in *in vivo* animal model	[68]
pH-sensitive poly(l-histidine)-based micelle system with folic acid	Enhancing cytoplasmic drug release due to proton-sponge effect of poly(l-histidine)	*In vivo* animal studies showed significant tumor regression effect in drug-resistant tumors	[69–73, 75]

**Table 2.** IC50 of PHSM^pop-upTAT^ and free DOX for human promyelocytic leukemia drug-resistant HL-60/MX2 (with decreased TopoII level), human promyelocytic leukemia HL-60 (with *bcl*-2), human lung drug-resistant NCI-H69/AR (with MRP), and human ovarian tumor A549 (with LRP) cells (n=9). All experiments were performed at pH 6.8 RPMI-1640/PBS medium. IC50 was obtained from the DOX concentration where 50% cell viability was achieved. Reproduced with permission from reference [73].

	**PHSM^pop-upTAT^**	**Free DOX**
**HL-60/MX2 a**	0.32 ± 0.07 μg/mL	1.12 ± 0.08 μg/mL
**HL-60 b**	0.10 ± 0.03 μg/mL	0.42 ± 0.07 μg/mL
**NCI-H69/AR c**	0.20 ± 0.06 μg/mL	0.75 ± 0.08 μg/mL
**A549 d**	0.75 ± 0.08 μg/mL	6.60 ± 0.09 μg/mL

a: IC50 after 1-hour incubation with DOX-loaded formulation;

b: IC50 after 1-hour incubation with DOX-loaded formulation;

c: IC50 after 24-hour incubation with DOX-loaded formulation;

d: IC50 after 48-hour incubation with DOX-loaded formulation.
